# Laparoscopic Surgery During Pregnancy: A Meta-Review and Quality Analysis Using the Assessment of Multiple Systematic Reviews (AMSTAR) 2 Instrument

**DOI:** 10.7759/cureus.63521

**Published:** 2024-06-30

**Authors:** Athanasios G Pantelis, Nikolaos Machairiotis, Sofoklis Stavros, Anastasios Potiris, Theodoros Karampitsakos, Dimitris P Lapatsanis, Petros Drakakis

**Affiliations:** 1 Surgical Department of Obesity and Metabolic Disorders, Athens Medical Group, Psychiko Clinic, Athens, GRC; 2 Third Department of Obstetrics and Gynecology, Attikon University Hospital, National and Kapodistrian University of Athens School of Medicine, Athens, GRC

**Keywords:** laparoscopic cholecystectomy, adnexal-ovarian laparoscopic surgery, laparoscopic cervical cerclage, laparoscopic appendectomy, amstar-2, fetal loss, surgical safety, pregnancy, minimally invasive surgery, laparoscopic surgery

## Abstract

Accumulation of experience with minimally invasive surgery over the last three decades has rendered laparoscopic surgery the mainstay of management for surgical pathology during pregnancy. In the present meta-review, we compiled the available evidence on the safety of laparoscopic and robotic-assisted surgeries during pregnancy, based on relevant systematic reviews (SR) and meta-analyses (MA). A systematic review was performed for articles published until February 2024 in English using PubMed/MEDLINE (Medical Literature Analysis and Retrieval System Online) and Google Scholar based on predefined selection and exclusion criteria. We implemented the Preferred Reporting Items for Systematic Reviews and Meta-Analyses (PRISMA) guidelines and included SRs and MAs examining women of childbearing age (population) who had undergone laparoscopic surgery or robotic-assisted laparoscopic surgery during pregnancy (intervention). The presence of comparison to open surgery was desirable but not mandatory (comparator). The included studies should necessarily report on fetal loss (outcome), and optionally on other metrics of fetal, maternal, or operative performance. We considered SRs/MAs analyzing randomized trials, observational studies, case reports, and case series (study design). The methodological quality of SRs/MAs not exclusively including case reports and case series was assessed with the Assessment of Multiple Systematic Reviews (AMSTAR) 2 instrument. A total of 1229 articles were screened, of which 78 were potentially eligible. Of these, 33 articles met our inclusion criteria, 18 containing SRs only and 15 SRs with MA. The examined disciplines were laparoscopic appendectomy (10 studies, 30.3%), laparoscopic cerclage for cervical insufficiency (eight studies, 24.2%), adnexal-ovarian laparoscopic surgery (five studies, 15.2%), laparoscopic cholecystectomy and biliary tree exploration (three studies, 9.1%), laparoscopic myomectomy (two studies, 6.1%), and one study each for laparoscopic surgery regarding pancreatic indications, adrenal indications, and bariatric complications (3.0%). The odds ratio/relative risk for fetal loss rate ranged from 0-1.9, with variable statistical significance depending on the discipline. Twenty-three out of the 33 studies were submitted to quality evaluation with the AMSTAR 2 instrument, with three being of “low quality” (13.0%) and the remaining 20 of “critically low quality” (87.0%). In conclusion, the widespread acceptance of laparoscopic surgery for treating surgical pathology during pregnancy is substantiated by heterogeneous and low-quality evidence. Literature mainly revolves around laparoscopic appendectomy, whereas other disciplines that may commonly arise during pregnancy, such as cholecystectomy and the acute abdomen following bariatric surgery, are underrepresented in the literature. Factors such as anatomical alterations that may affect surgical access, surgeon’s expertise, and the biological course of the underlying pathology should be taken into consideration when selecting the appropriate mode of operating during pregnancy.

## Introduction and background

Non-obstetric surgery during pregnancy might be required in up to 2% of pregnancies, with the common indications including emergency conditions and malignancies [1]. Regardless of the increased frequency at which these procedures are being performed, there are still concerns revolving around potential teratogenesis induced by anesthetic agents, early pregnancy loss, preterm birth, and hypoxic fetal injury [2,3]. Additionally, anatomical and physiological alterations that occur during pregnancy might compromise the health of the maternal-fetal unit or obscure the perioperative monitoring, including the physiologic anemia of pregnancy, respiratory alkalosis, change of the relative visceral anatomy due to the expanding uterus, increased portal pressure, increase in liver enzymes and dilution of plasma proteins, increased glomerular filtration, and alterations in coagulation factors, to name a few [2,4].

With accumulating experience over the years, laparoscopic surgery has gained popularity as the preferred modality for elective and emergency non-trauma surgery during pregnancy [4]. Several national guidelines deem laparoscopy during pregnancy a feasible and safe modality [5]. In the most comprehensive guideline (227 references), originally released by the Society of American Gastrointestinal and Endoscopic Surgeons (SAGES) in 2017 and updated in 2023, laparoscopic treatment of acute abdominal disease seems to offer similar benefits to pregnant and non-pregnant women compared to open surgery, while laparoscopy can be performed at any trimester, provided there are adequate surgical indications [6]. Notably, both statements received strong recommendations based on moderate quality of evidence.

The present meta-review aims to compile systematic reviews (SRs) and/or meta-analyses (MAs) on laparoscopic surgery during pregnancy on any indication, with a primary focus on safety and a secondary focus on effectiveness. Additionally, we scrutinized the methodological quality of relevant studies with the use of Assessment of Multiple Systematic Reviews (AMSTAR) 2, a critical appraisal tool for SRs including randomized and non-randomized studies of healthcare interventions [7].

## Review

Methods

Search Strategy

Two authors (AGP, NM) conducted a systematic search within two scientific electronic databases, PubMed/MEDLINE (Medical Literature Analysis and Retrieval System Online) and Google Scholar, according to the updated Preferred Reporting Items for Systematic Reviews and Meta-Analyses (PRISMA) statement [8]. The search terms used were ‘laparoscop*” and “pregnancy”, whereas additional filters were implemented regarding the years of publication (1990-2024) and the type of publication (systematic review OR meta-analysis). The references of the included SRs and MAs were searched for further relevant citations. An initial search was undertaken in October 2023 and a repeat search was performed in March 2024 prior to final submission. Studies were limited to those published in the English language. The detailed search strategy can be found in Appendix A.

Inclusion and Exclusion Criteria

We implemented the PICOS (Patient, Intervention, Control (comparator), and Outcome) process as a guidance through the inclusion criteria:

Population: We included SRs and MAs analyzing women of childbearing age who had undergone any laparoscopic procedure during any trimester of pregnancy, including (but not limited to) cholecystectomy, appendectomy, solid organ resection, surgery for adnexal masses, and adnexal torsion. We also included SRs and/or MAs referring to laparoscopic cerclage, given the increasing incidence of this procedure during pregnancy and the abundance of relevant literature. In case an SR/MA examined more than one discipline, we classified it as “Multiple”. Finally, we included SRs/MAs that contained robotic-assisted laparoscopic surgery in pregnancy, with the rationale that the principles of minimally invasive access and the sequelae of pneumoperitoneum remain the same.

Intervention: The respective reviews should have included patients who had undergone laparoscopic surgery or robotically-assisted laparoscopic surgery in order to be considered for inclusion.

Comparator: The existence of a comparator group (i.e., open surgery) was desirable but not mandatory for a study to be included in our analysis. A comparator group was expected to be found in MAs but not necessarily in SRs.

Outcome: It was mandatory for a study to report fetal loss as an outcome in order to be considered for inclusion. Additionally, any other reported outcomes, concerning fetal (i.e., preterm birth, birth weight, Apgar score, etc.), maternal (maternal mortality, pre-eclampsia/eclampsia, other maternal complications, etc.), or operative (i.e., operative time, elective or emergency cesarian section, blood loss, hospital stay, etc.) issues were also documented and included in our analyses. Forest plots for outcomes reported in three or more MAs were devised for purposes of comparative analysis (rather than yielding pooled results).

Study design: We considered SRs with or without MA, which in turn could potentially have included randomized controlled trials (RCTs) and non-randomized studies of interventions (NRSIs, i.e., prospective cohort studies, retrospective cohort studies, registries, etc.). Notably, we also considered SRs containing case reports and/or case series, particularly for rare diseases and disciplines. We further performed a quality analysis of all the included SRs and MAs.

For the purposes of our study, we focused on abdominal visceral surgery occurring during pregnancy, including surgery to the female reproductive organs, i.e., for fibroids, adnexal cysts, or cervical cerclage. However, we did not include SRs/MAs on laparoscopy for managing ectopic pregnancy, as this essentially would lead to non-viable pregnancy which was outside the scope of our analysis. Narrative reviews, conference abstracts, and reviews examining patients in whom laparoscopy and pregnancy did not coincide (i.e., those investigating the childbearing potential of patients who had undergone laparoscopy) were also excluded. The same was applicable to studies for which the full text was not available after exhaustive search in institutional libraries and personal contact with corresponding authors via e-mail or through the ResearchGate website (www.researchgate.net), or for which the full text was not in English.

The two authors mentioned earlier screened titles and abstracts and consequently examined the full text of eligible studies or studies ambiguous for inclusion. In case of disagreement, further review of inclusion and exclusion criteria was performed, and a third author (DPL) served the role of final arbitrator. Duplicate merging and referencing were performed with the aid of Zotero reference management software (Corporation for Digital Scholarship, Vienna, Virginia, United States).

Data Extraction and Synthesis

For the purpose of data extraction, we scrutinized the entire paper, including the full-text and supplementary material, when available. After achieving consensus on the SRs/MAs that would be included in our analysis, data extraction was performed in duplicate (AGP, NM) and the following data were registered in MS Excel spreadsheet (Microsoft Corporation, Redmond, Washington, United States): first author, year of publication, country (/-ies) of origin, doi number, discipline, search period, search database(s), inclusion criteria, exclusion criteria, review method (whether the study was SR only or SR with MA), number of included studies in SR, number of included studies in MA, number of patients in SR, number of patients in MA, comparator (i.e., open abdominal or transvaginal surgery), number of patients in laparoscopic group (also including patients who had undergone robotic-assisted laparoscopic surgery), gestational age at laparoscopic surgery, number of patients in comparator group, gestational age at comparator group, outcomes, and for MA specifically, odds ratio (OR), relative risk (RR), or mean difference (MD) depending on the reported measure(s), 95% confidence interval (95%CI), p value for OR/RR/MD, *I^2^* (%) value for heterogeneity, and p value for *I^2^*. A p-value of <0.05 was considered significant, whereas *I^2^* <30% was indicative of low heterogeneity, 30% ≤*I^2^* ≤50% indicated moderate heterogeneity, and *I^2^* >50% represented high heterogeneity. Major findings were qualitative for SRs and quantitative for MAs. Additionally, we utilized the GROOVE (Graphical Representation of Overlap for Overviews) tool to assess and visualize the overlap of primary studies among MAs with the same subject (https://es.cochrane.org/es/groovetool) [9]. According to this tool, the overlap among primary studies is considered slight when it is <5%, moderate if 5-10%, high if 10-15%, and very high if it is equal to or greater than 15%.

Quality Assessment

We implemented the AMSTAR 2 instrument to evaluate the methodological quality of the included studies. This tool has been developed to thoroughly appraise SRs and MAs containing RCTs and/or NRSIs. Consequently, we excluded SRs/MAs containing only case reports and/or case series from the scrutiny of the AMSTAR 2 instrument.

The AMSTAR 2 instrument includes 16 items in total, critical (items 2, 4, 7, 9, 11, 13, and 15) and non-critical ones. Each item may take two values, 1 for yes (item covered in the examined SR/MA) and 0 for no (item not examined). Additionally, some items may take the value 0.5 in case of partial coverage of the respective object. Based on this grading, each SR/MA gets a final quality assessment (high: 0-1 non-critical flaw, moderate: >1 non-critical flaw, low: 1 critical flaw with/without non-critical flaws, critically low: >1 critical flaw, regardless of non-critical flaws). The purpose of this tool is not to attribute a cumulative score to each study, given the different gravity of each item, but to yield a final rating based on how many crucial and non-crucial items are covered by a given SR/MA. Nevertheless, there are publications that estimate a total score for purposes of comparability [10-12], and this is what we did in the meta-review in hand. We used the online checklist calculator that is available to yield the AMSTAR 2 score for each study (https://amstar.ca/Amstar_Checklist.php).

Identical to the process of data extraction, we scrutinized both the full text and supplementary material (wherever available) to assess each item of the AMSTAR 2 tool. We compiled the respective scores and ratings in a comprehensive table and visualized the examined SRs and MAs in a pyramid diagram according to their level of evidence (i.e., containing NRSIs only, both NRSIs and RCTs, or RCTs only).

Results

Study Characteristics

**Figure 1:**
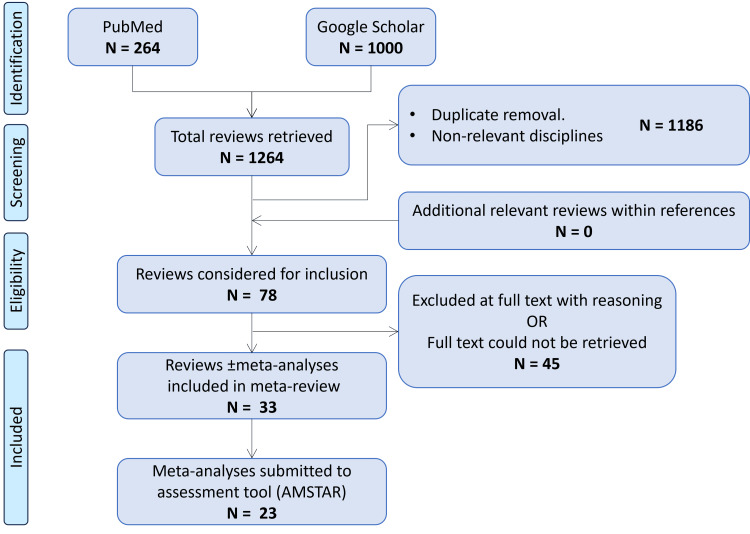
PRISMA-based flowchart for study selection PRISMA: Preferred Reporting Items for Systematic Reviews and Meta-Analyses

We screened 1264 articles in total (PubMed: 264, Google Scholar: 1000). In Google Scholar, we screened the first 1000 citations, given that the relevance of the appearing articles decreased exponentially after the first 300 citations. After the removal of duplicates, non-review articles, and articles with non-relevant disciplines, there were 78 papers left, the abstracts of which were screened. Forty-five articles were excluded with reasoning (narrative reviews, laparoscopy not performed during pregnancy, etc.) or because the full text could not be retrieved after searching thoroughly through several libraries and attempting to contact the authors (Appendix B). Eventually, we included 33 articles in our meta-review, 18 SR-only [13-30] and 15 SRs with MA [31-45]. Among them, 23 articles contained non-case report/ non-case series NRSIs or RCTs and were subsequently evaluated with the AMSTAR 2 instrument [13-16,18-20,30-45]. Figure 1 illustrates the flowchart of study selection.

**Figure 2:**
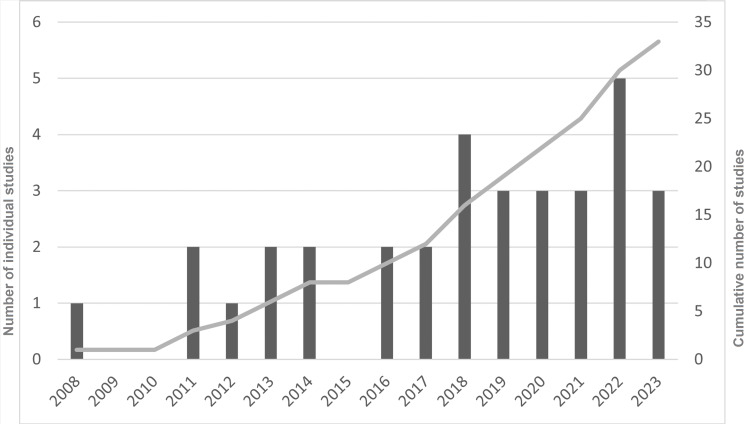
Temporal distribution of the included review studies. The left y-axis represents number of individual studies and corresponds to the dark grey vertical bars.  The right y-axis represents cumulative number of studies and corresponds to the light grey continuous line.

The oldest retrieved study dates back to 2008, with a progressive increase of publications being evident over time and more than 75% of the included studies having been published after 2015 (Figure 2).

**Figure 3:**
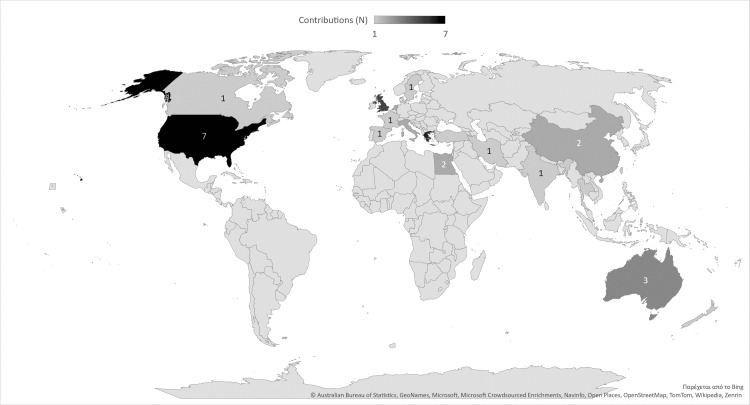
Geographical distribution of the included review studies.

Regarding geographical distribution, the most common countries of origin were the United States (appearing in seven reviews), Greece (six reviews), and the UK (five reviews) (Figure 3). 

**Figure 4:**
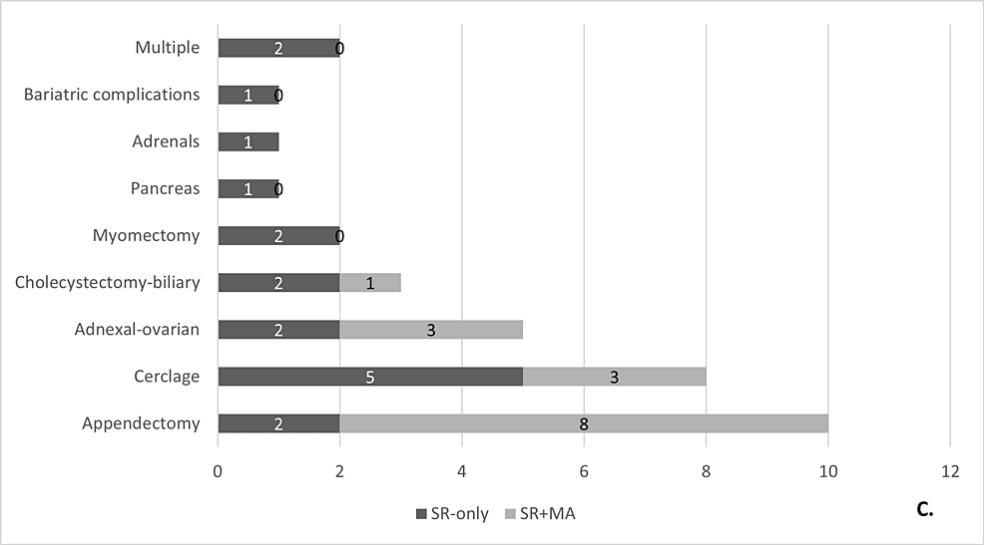
Distribution of the included review studies by discipline. SR: systematic review; MA: meta-analysis

Based on their discipline, the studies were classified as follows, in descending order: LA 10 studies (30.3%) [13,14,31-38], laparoscopic cerclage for cervical insufficiency eight studies (24.2%) [15-19,39-41], adnexal-ovarian laparoscopic surgery five studies (15.2%) [20,21,42-44], laparoscopic cholecystectomy and biliary tree exploration three studies (9.1%) [22,23,45], laparoscopic myomectomy two studies (6.1%) [24,25], and one each for laparoscopic surgery regarding pancreatic indications [28], adrenal indications [26], and bariatric complications (3.0%) [27]. Two studies were interdisciplinary, one examining robotic-assisted laparoscopic surgery during pregnancy for any indication [29], and one investigating non-obstetric surgery during pregnancy in general [30]. Figure 4 summarizes the distribution of the included studies by discipline. 

**Figure 5:**
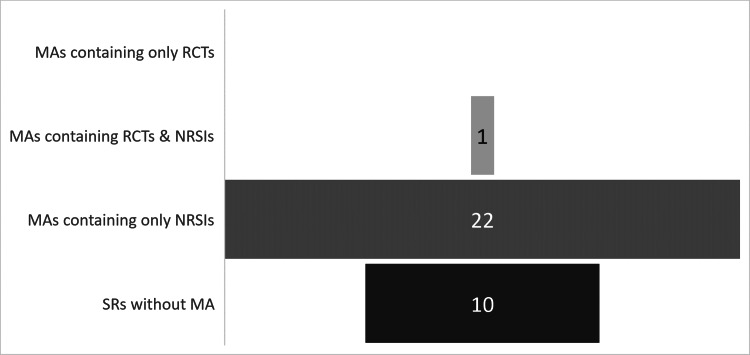
Distribution of the included review studies by level of evidence. SR: systematic review; MA: meta-analysis; RCT: randomized controlled trial; NRSI: non-randomized studies of intervention

The majority of the included reviews analyzed observational studies (23 reviews, 69.7%), whereas 10 reviews (30.3%) contained only case reports and case series. Notably, there was only one study within the discipline of laparoscopic cervical cerclage that contained one RCT along with 42 observational studies [41]. The distribution of the reviews according to the level of evidence of the included studies is depicted in Figure 5.

Intervention Effects

LA: This category was the most abundant, containing 10 studies (two SR-only [13,14] and eight SRs with MA) [31-38], and spanning the period from 2008 to 2022. Two reviews contained mixed observational studies and case reports [13,14].

**Figure 6:**
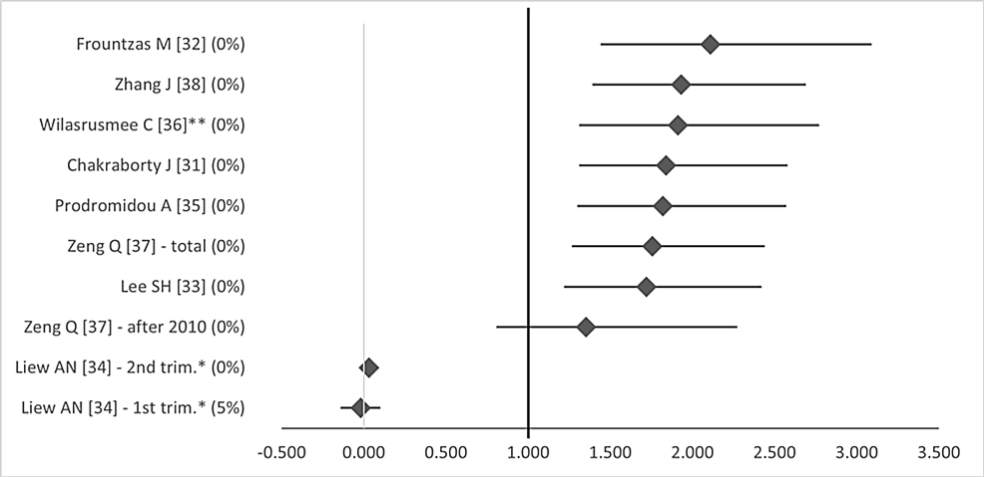
Forest plot comparing fetal loss between open and laparoscopic appendectomy. Values on the right side favor open appendectomy, whereas those to the left favor laparoscopic appendectomy. All values represent odds ratio, except those with (*), which represent risk difference, and those with (**), which represent relative risk. Numbers in brackets indicate the respective study reference number [31-38].

Regarding fetal outcomes, the two SRs report that LA "during pregnancy might be associated with higher rates of fetal loss” [13], with a significant rate of fetal loss following LA for complicated appendicitis compared to non-complicated appendicitis [14]. Of note, the latter study was conducted before 2010. Besides, seven out of eight MAs estimated the pooled OR for fetal loss, which ranged from 0.57 to 2.11. Six of them featured no heterogeneity [31-33,35-37], whereas one had medium heterogeneity (*I^2^* = 38%) [38]. Six studies showed a significant increase in fetal loss with LA compared to open appendectomy (OA) (OR 1.72-2.11, 95%CI 1.22-3.09) [31-33,35,36,38]. However, Zeng et al. demonstrated that the difference in fetal loss between OA and LA was eliminated when examining cases performed after 2010 (OR 0.74, 95%CI 0.44-1.24) [37]. Additionally, Liew et al. measured the risk difference for fetal loss (instead of OR) and found no significant difference between LA and OA in both the first and second trimesters [34]. Figure 6 is the forest plot that summarizes the differences between LA and OA regarding fetal loss. 

**Figure 7:**
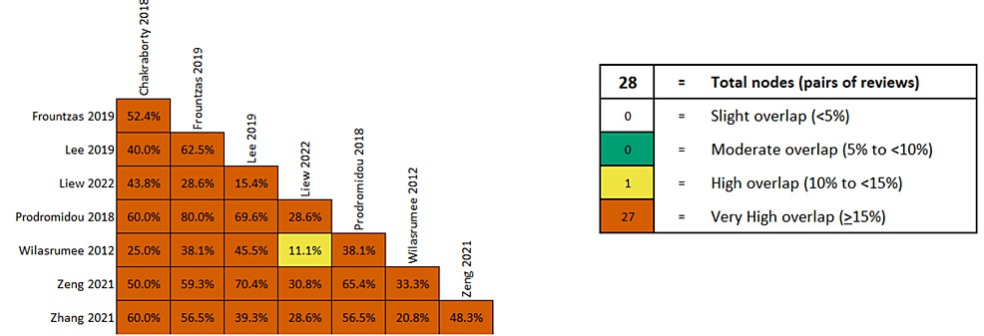
Overlap of studies on fetal loss after laparoscopic appendectomy during pregnancy, according to the GROOVE tool. References: [31-38]

The overlap of primary studies was very high, according to the GROOVE tool (Figure 7). 

**Figure 8:**
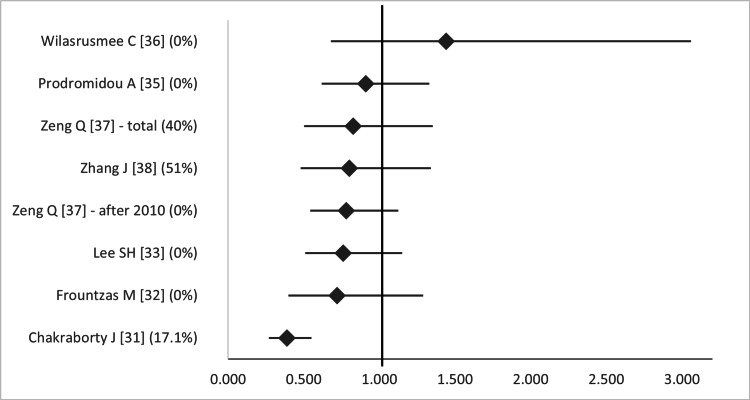
Forest plot comparing preterm birth between open and laparoscopic appendectomy. Values on the right side favor open appendectomy, whereas those to the left favor laparoscopic appendectomy. All values represent odds ratio. Numbers in brackets indicate the respective study reference number [31-33,35-38].

**Figure 9:**
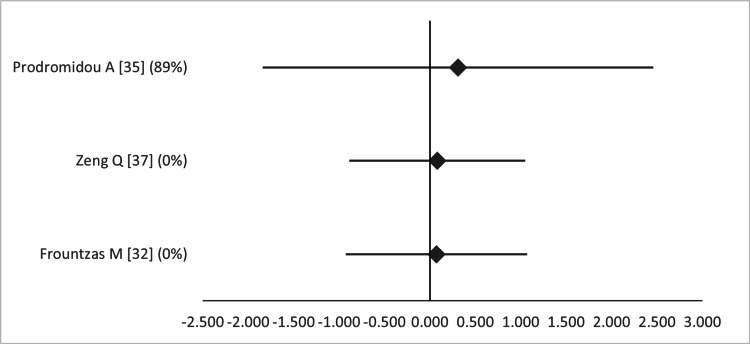
Forest plot comparing birth weight between open and laparoscopic appendectomy. Values on the right side favor open appendectomy, whereas those to the left favor laparoscopic appendectomy. All values represent mean difference. Numbers in brackets indicate the respective study reference number [32,35,37].

**Figure 10:**
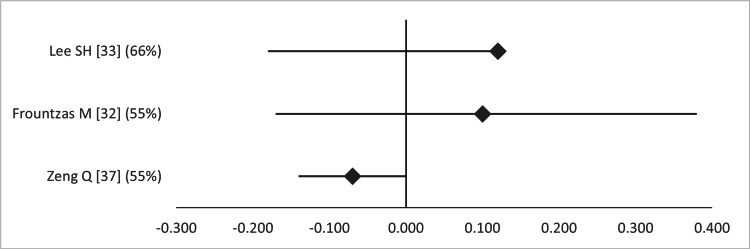
Forest plot comparing Apgar score at one minute between open and laparoscopic appendectomy. Values on the right side favor open appendectomy, whereas those to the left favor laparoscopic appendectomy. All values represent mean difference. Numbers in brackets indicate the respective study reference number [32,33,37].

On the other hand, only one MA showed a significant reduction in the OR of preterm births after LA (Figure 8) [31], whereas there was no significant change in the mean difference between LA and OA in terms of birth weight (Figure 9) and Apgar score at one minute (Figure 10).

**Figure 11:**
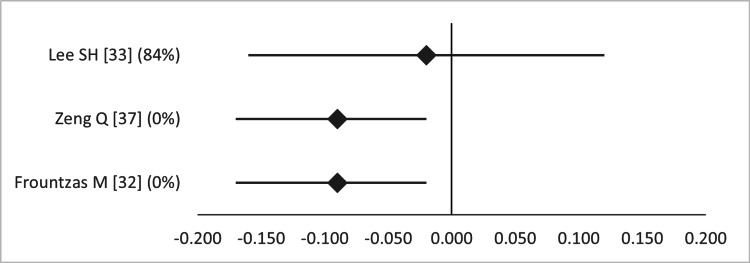
Forest plot comparing Apgar score at five minutes between open and laparoscopic appendectomy. Values on the right side favor open appendectomy, whereas those to the left favor laparoscopic appendectomy. All values represent mean difference. Numbers in brackets indicate the respective study reference number [32,33,37].

Interestingly, two out of three studies found a significant difference in Apgar score at five minutes favoring LA (Figure 11) [32,37]. The overlap of primary studies was very high for all these meta-analyses, according to the GROOVE tool (graphs available upon request).

**Figure 12:**
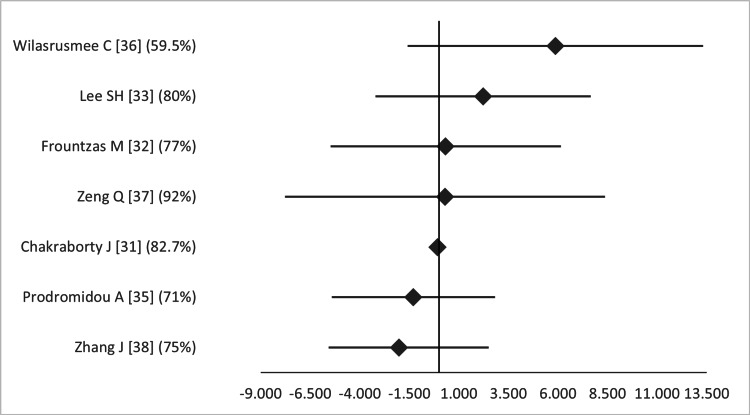
Forest plot comparing operative time between open and laparoscopic appendectomy. Values on the right side favor open appendectomy, whereas those to the left favor laparoscopic appendectomy. All values represent mean difference. Numbers in brackets indicate the respective study reference number [31-33,35-38].

Regarding perioperative parameters, there was no significant difference in operative time according to seven MAs that examined this item, with the reservation that all of these sub-analyses featured high heterogeneity (*I^2^* 59.5-92%) (Figure 12) [31-33,35-38]. 

**Figure 13:**
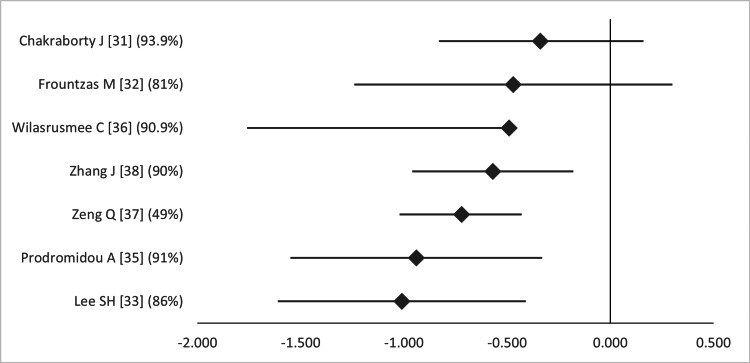
Forest plot comparing hospital length of stay between open and laparoscopic appendectomy. Values on the right side favor open appendectomy, whereas those to the left favor laparoscopic appendectomy. All values represent mean difference. Numbers in brackets indicate the respective study reference number [31-33,35-38].

Moreover, hospital length of stay (LoS) was significantly shorter in five out of seven MAs and non-significantly in the remaining two (Figure 13) [31-33,35-38]. However, the heterogeneity in this regard was high (*I^2^* = 81.0-93.9%). The overlap was also very high, according to the GROOVE tool (graphs available upon request).

**Figure 14:**
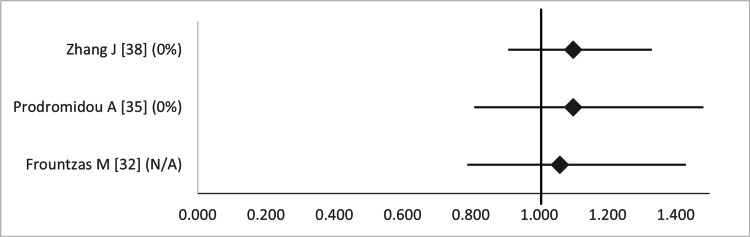
Forest plot comparing cesarean section between open and laparoscopic appendectomy. Values on the right side favor open appendectomy, whereas those to the left favor laparoscopic appendectomy. All values represent odds ratio. Numbers in brackets indicate the respective study reference number [32,35,38].

Eventually, when considering maternal outcomes, there was no significant impact on the rates of cesarean section, according to three studies that examined this parameter (Figure 14) [32,35,38]. 

**Figure 15:**
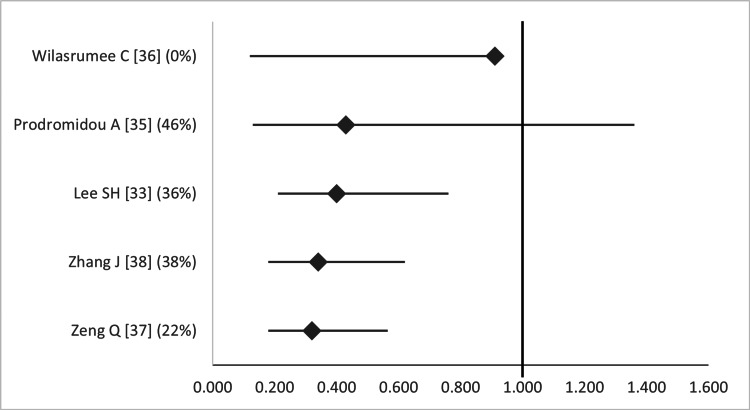
Forest plot comparing superficial surgical site infection (wound infection) between open and laparoscopic appendectomy. Values on the right side favor open appendectomy, whereas those to the left favor laparoscopic appendectomy. All values represent odds ratio. Numbers in brackets indicate the respective study reference number [33,35-38].

Regarding superficial surgical site infections (SSIs), LA was associated with a significant reduction of wound infections in four out of five studies [33,36-38], and a non-significant reduction in one (Figure 15) [35]. 

**Figure 16:**
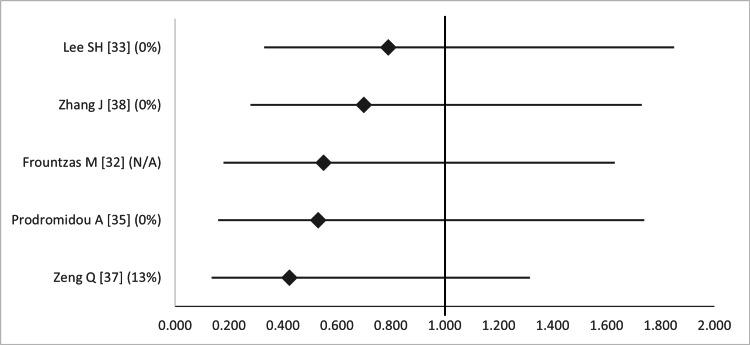
Forest plot comparing deep surgical site infection (intra-abdominal abscess) between open and laparoscopic appendectomy. Values on the right side favor open appendectomy, whereas those to the left favor laparoscopic appendectomy. All values represent odds ratio. Numbers in brackets indicate the respective study reference number [32,33,35,37,38].

Finally, when considering deep SSIs (intra-abdominal abscess), LA was associated with a decreased rate as compared to OA, but this finding was non-significant in all five studies that examined this item (Figure 16) [32,33,35,37,38]. The overlap was also high, according to the GROOVE tool (graphs available upon request).

Laparoscopic cervical cerclage: The indication of cervical cerclage placement is cervical shortening that might lead to adverse gestational outcomes heralded by spontaneous preterm birth. Cervical cerclage might be performed transvaginally, transabdominally in an open fashion (TAC), or laparoscopically. Ideally, it should be placed before conception, but it can be placed during pregnancy as a rescue procedure [46]. For the purposes of our study, we focused on reviews that included the investigation of the role of laparoscopic cervical cerclage placement during pregnancy, either exclusively or in conjunction with other methods (transvaginal, TAC) or different timing (before pregnancy).

Our search yielded eight studies, five SRs-only [15-19] and three SRs with MA [39-41], from 2011 to 2022. For one MA, forest plots could not be retrieved, neither in the main text nor in the supplementary file, as such pertinent data could not be retrieved and incorporated in the cumulative analysis [39].

**Figure 17:**
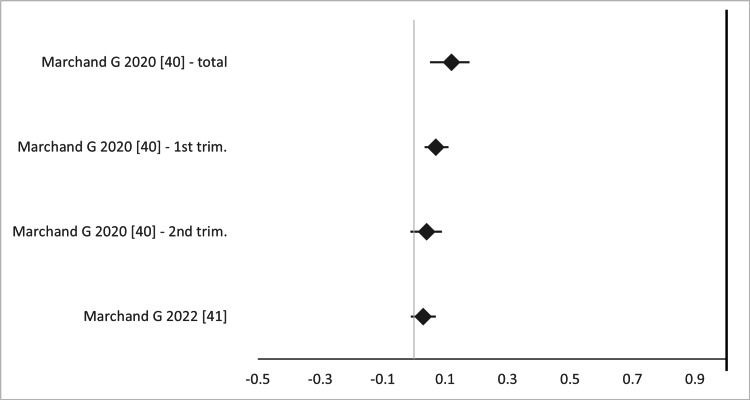
Forest plot comparing fetal loss between open (transabdominal) and laparoscopic cervical cerclage. Values on the right side favor open cervical cerclage, whereas those to the left favor laparoscopic cervical cerclage. All values represent risk ratio. Numbers in brackets indicate the respective study reference number [40,41].

**Figure 18:**
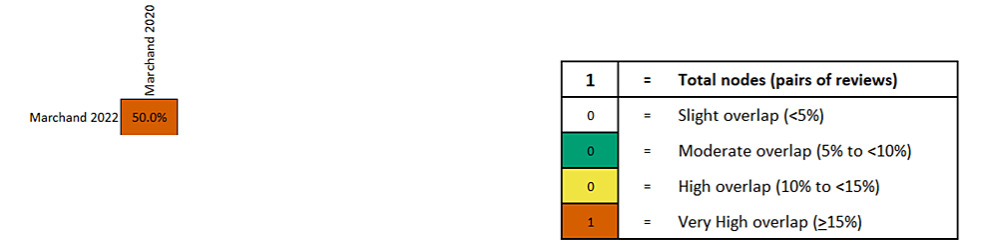
Overlap of studies on fetal loss after laparoscopic cervical cerclage during pregnancy, according to the GROOVE tool. References: [40,41].

In the five SRs and one MA, the rate of fetal loss ranged from 0% to 0.13% in the laparoscopic cerclage (LC) group and from 0% to 13% in the transabdominal (wherever data is available) (p 0.730->0.99) [15-19,39]. Marchand et al. have published two MAs on the subject and have shown that LC is safe during pregnancy, at both the first and second trimester and overall (OR 0.03-0.12, 95%CI (-0.01)-0.178) (Figure 17). The overlap among primary studies was very high (Figure 18).

Preterm premature rupture of membranes (PPROM) was investigated in two reviews (0-0.055% for LC and 0.007-0.063 for TAC, p >0.05) [15, 39], and one MA (OR 0.030, 95%CI 0.009-0.053) [40]. Operative time ranged from 52.52 to 307 minutes for LC and from 34.22 to 70.8 minutes for TAC, with variable significance [15-17,39,41]. No other meaningful comparison could be made among studies because no other metric was investigated in more than two studies.

Adnexal-ovarian laparoscopic surgery: Our search yielded four reviews of articles examining the impact of laparoscopic surgery during pregnancy with the indication of adnexal and ovarian pathology. Among them, three MAs were investigating adnexal masses (spanning the period 2016-2021) [42-44], one SR for ovarian tumors [20], and one SR for adnexal torsion [21].

**Figure 19:**
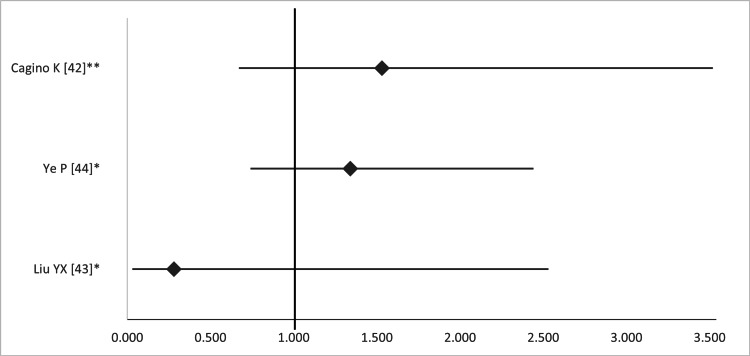
Forest plot comparing fetal loss between open and laparoscopic ovarian-adnexal surgery. Values on the right side favor open surgery, whereas those to the left favor laparoscopic surgery. All values represent relative risk (*) or odds ratio (**). Numbers in brackets indicate the respective study reference number [42-44].

According to the three MAs, the OR or RR of fetal loss ranged from 0.28 to 1.53, but this finding was not significant in any study (Figure 19). 

**Figure 20:**
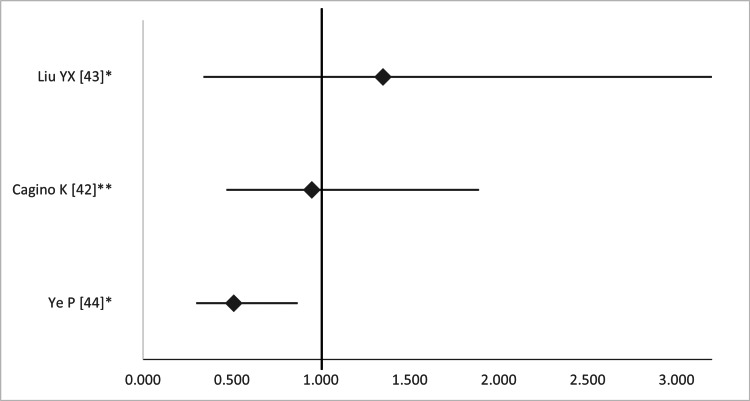
Forest plot comparing preterm birth between open and laparoscopic ovarian-adnexal surgery. Values on the right side favor open surgery, whereas those to the left favor laparoscopic surgery. All values represent relative risk (*) or odds ratio (**). Numbers in brackets indicate the respective study reference number [42-44].

All three studies also investigated the risk of preterm birth. Two of them found a non-significant association between laparoscopic surgery and preterm birth [42,43], whereas one showed a benefit with laparoscopic surgery (OR 0.51, 95%CI 0.34-5.38) (Figure 20) [44]. 

**Figure 21:**
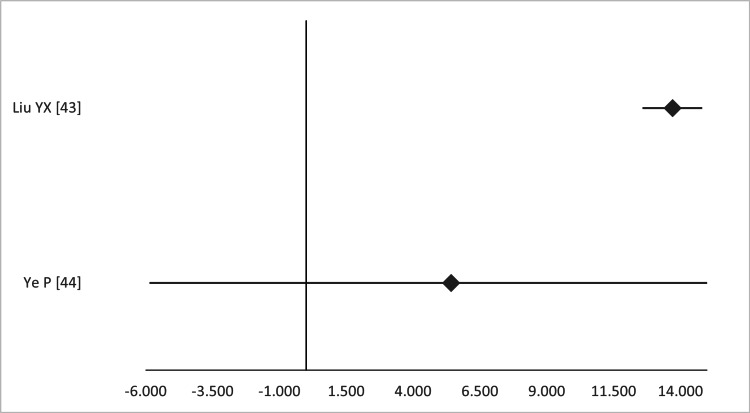
Forest plot comparing operative time between open and laparoscopic ovarian-adnexal surgery. Values on the right side favor open surgery, whereas those to the left favor laparoscopic surgery. All values represent relative risk. Numbers in brackets indicate the respective study reference number [43,44].

Findings regarding operative time were conflicting: one study found a significant increase with laparoscopy [43], one found a non-significant increase [44], and showed a non-significant difference in operative time (80 versus 72.5 minutes, p = 0.09, Figure 21) [42]. 

**Figure 22:**
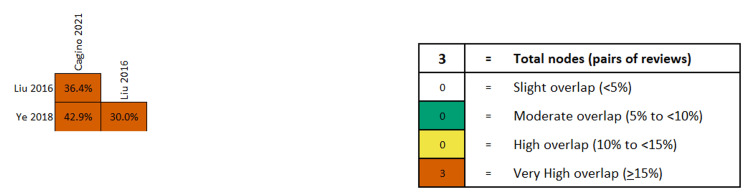
Overlap of studies on fetal loss after laparoscopic ovarian-adnexal surgery during pregnancy, according to the GROOVE tool. References: [42-44]

The overlap among primary studies in all meta-analyses was very high, according to the GROOVE tool (Figure 22 for fetal loss, rest of graphs available upon request). No other meaningful comparison could be made based on the provided data. 

Laparoscopic cholecystectomy & bile duct exploration: This category included one meta-analysis comparing laparoscopic cholecystectomy (LC) versus open cholecystectomy during pregnancy [45], one SR on LC [23], and one SR on laparoscopic bile duct exploration (LBDE) during pregnancy [22]. The MA found a cumulative OR of 0.39 (95%CI 0.07-2.19) regarding fetal loss, whereas the respective figures were a non-significant 1.35 (95%CI 0.41-5.14) for preterm delivery, a significant 0.45 (95%CI 0.25-0.82) for perioperative complications, and a non-significant 3.88 (95%CI 0.15-100.23) for maternal mortality [45]. The SR on LC retrieved 590 relevant cases, with a fetal loss rate of 0.4%, a preterm delivery rate of 5.7%, and a postoperative complication rate of 4% [23]. Finally, the literature review on LBDE identified 15 cases (among which seven had been published in the past), with a success rate of the procedure reaching 86%, without major fetal or maternal complications [22].

Laparoscopic myomectomy: We retrieved two systematic reviews of case reports and series negotiating the topic of laparoscopic myomectomy during pregnancy [24,25]. There were no fetal losses or preterm births mentioned in any of the included cases.

Single-reference disciplines: There was one study retrieved in each of the following disciplines: adrenals [26], bariatric complications [27], and pancreas [28].

The review on adrenals comprised six laparoscopic cases and one robotic for pheochromocytoma, without any mention of maternal or fetal death among these specific cases that were treated with minimally invasive procedures [26].

The study on bariatric complications was a systematic review of case reports and series that contained 19 attempted laparoscopic intrapartum operations, mostly for intussusception or internal hernia [27]. Among them, 10 were accomplished laparoscopically and the rest were converted to open surgery. Among these laparoscopic cases, there were no maternal deaths but there were two fetal losses (20%), versus 2.5% maternal deaths and 7.5% fetal losses in the entire cohort of cases.

The study on the pancreas was about pancreatic cystic neoplasms in pregnancy [28]. It described one case of laparoscopic distal pancreatectomy with an uneventful postoperative course, which also contained a review that yielded 47 published cases of open surgery.

Multidisciplinary studies: Our search yielded two studies that spanned across more than one discipline regarding laparoscopic surgery during pregnancy. The one referred to non-obstetric robotic-assisted laparoscopic surgery and yielded 11 studies with 38 cases, 33 regarding ovarian pathologies and five regarding urologic issues [29]. There were no documented fetal losses in either arm, and there was one preterm birth documented in each group (ovarian and urologic). The ovarian group also entailed one case of PPROM, one cesarean section, and one postoperative complication. The respective numbers for the urologic group were zero PPROMs, three cesarean sections, and zero postoperative complications.

The other multidisciplinary study was on non-obstetric surgery during pregnancy in general, irrespective of the type of operation [30]. It included studies on appendectomy, cholecystectomy, abdominal surgery in general, non-obstetric gynecologic surgery, trauma, and orthopedic surgery. Among 32 studies and 36,120 patients, they found a prevalence of 47% (range 5-89%) for laparoscopic surgery versus 53% for open. Based on data from nine studies and 693 patients, the OR of laparoscopy for miscarriage was 1.9 (95%CI 0.81-4.3), while the respective figure for preterm birth was 0.68 (95%CI 0.58-0,79), for low birth weight 0.70 (95%CI 0.5-1.1), Apgar score <7 at five minutes 0.50 (95%CI 0.2-1.1), and maternal mortality 6.60 (95%CI 4.0-11.1).

Methodological Quality

As mentioned earlier, 23 of the 33 studies fulfilled the criteria to be assessed with the AMSTAR 2 instrument regarding their methodological quality. Among the eligible studies, the mean AMSTAR 2 score was 7.23 and the median was 11.5. Three studies were characterized as “low” quality (one critical item missing) and the rest 20 as “critically low” (more than one critical item missing). No “moderate” or “high” quality studies were retrieved, according to the AMSTAR 2 instrument. The most frequently missing items were item 2 (protocol had not been registered before the commencement of the study or no reference on protocol was made in the study) and 7 (no justification or list provided for the excluded studies). The distribution of the quality of studies by discipline was as follows: appendectomy - nine critically low, one low; laparoscopic cervical cerclage - six critically low, one low; laparoscopic adnexal/ovarian surgery - three critically low, one low; cholecystectomy - one critically low; multidisciplinary studies - one critically low. Table 1 contains the rating of the included studies according to the AMSTAR 2 instrument in detail.

**Table 1:** Rating of the included review studies according to the AMSTAR 2 instrument. *The study indicates "meta-analysis" but it does not contain one. **Study protocol mentions risk of bias (RoB), but no such analysis could be retrieved. ***AMSTAR 2 score: minimum = 0; maximum = 12.5, mean = 7.23; median = 10.5. AMSTAR: Assessment of Multiple Systematic Reviews

Discipline	First author	Year	Design of included studies	1	2	3	4	5	6	7	8	9	10	11	12	13	14	15	16	Sum	AMSTAR 2 Assessment
Appendectomy	Walker et al. [13]	2014	25 case reports + cohort	0	0	1	0	0	0	0	0.5	0	0	0	0	0	0	0	1	2.5	Critically low
Appendectomy	Walsh et al. [14]	2008	22 case reports + cohort	0	0	0	0	0	0	0	0.5	0	0	0	0	0	0	0	1	1.5	Critically low
Cerclage	Burger et al. [15]	2011	observational (retrospective)	1	0	0	0.5	1	1	0	1	0	0	0	0	0	0	0	0	4.5	Critically low
Cerclage	El-Nashar et al. [16]	2013	observational	0	0	0	0	0	0	0	1	0	0	0	0	0	0	0	0	1	Critically low
Cerclage	Iavazzo et al. [17]	2018	case reports & case series	-	-	-	-	-	-	-	-	-	-	-	-	-	-	-	-	-	-
Cerclage	Moawad et al. [18]	2017	observational & case-controls	1	0	0	0.5	1	0	0	0.5	0	0	0	0	0	0	0	0	3	Critically low
Cerclage	Tulandi et al. [19]	2014	observational & case-controls	1	0	0	0.5	1	0	0	0.5	0	0	0	0	0	0	0	1	4	Critically low
Adnexal-ovarian	Aggarwal and Kehoe [20]	2011	observational (27 retro + 6 pro)	1	0	0	0	0	0	0	0	0	0	0	0	0	0	0	0	1	Critically low
Adnexal-ovarian	Didar et al. [21]	2023	case reports & case series	-	-	-	-		-	-	-	-	-	-	-	-	-	-	-	-	-
Cholecystectomy-biliary	Lopez-Lopez et al. [22]	2023	case reports & case series	-	-	-	-	-	-	-	-	-	-	-	-	-	-	-	-	-	-
Cholecystectomy-biliary	Nasioudis et al. [23]	2016	case reports & case series	-	-	-	-	-	-	-	-	-	-	-	-	-	-	-	-	-	-
Myomectomy	Diakosavvas et al. [24]	2022	case reports & case series	-	-	-	-	-	-	-	-	-	-	-		-	-	-	-	-	-
Myomectomy	Spyropoulou et al. [25]	2020	case reports & case series	-	-	-	-	-	-	-	-	-	-	-	-	-	-	-	-	-	-
Adrenals	Biggar and Lennard [26]	2013	case reports & case series	-	-	-	-	-		-	-	-	-	-	-	-	-	-	-	-	-
Bariatric complications	Petrucciani et al. [27]	2019	case reports & case series	-	-	-	-	-		-	-	-	-	-	-	-	-	-	-	-	-
Pancreas	Fogliati et al. [28]	2022	case reports & case series	-	-	-	-	-	-	-	-	-	-	-	-	-	-	-	-	-	-
Multiple	Capella et al. [29]	2020	case reports & case series	-	-	-	-	-		-	-	-	-	-	-	-	-	-	-	-	-
Multiple	Haataja et al. [30]	2023	observational (58 retro + 2 pro)	1	0	0	0.5	1	1	0	1	0	0	0	0	0	1	0	1	6.5	Critically low
Appendectomy	Chakraborty et al. [31]	2018	observational (16 retro + 1 pro)	1	0	0	0.5	1	1	0	1	0	0	1	0	0	0	1	1	7.5	Critically low
Appendectomy	Frountzas et al. [32]	2019	observational (retro + pro)	1	1	0	1	0	0	0	0.5	1	0	1	0	1	0	0	0	6.5	Critically low
Appendectomy	Lee et al. [33]	2019	observational (10 retro + 1 pro)	1	0	0	0.5	1	1	0	0.5	0	0	1	0	0	1	1	1	8	Critically low
Appendectomy	Liew et al. [34]	2022	observational (8 retro + 1 pro)	0	0	0	0.5	1	1	0	0.5	0	0	0	0	0	1	0	1	5	Critically low
Appendectomy	Prodromidou et al. [35]	2018	observational (19 retro + 1 pro)	1	0	0	1	0	0	1	1	1	0	1	0	0	0	0	1	7	Critically low
Appendectomy	Wilasrusmee et al. [36]	2012	observational (3 retro + 8 pro)	1	0	0	0.5	1	1	0	0.5	1	0	1	1	1	1	1	1	11	Critically low
Appendectomy	Zeng et al. [37]	2021	observational (25 retro + 2 pro)	1	0	0	0.5	1	1	0	0.5	1	0	1	1	1	1	1	0	10	Critically low
Appendectomy	Zhang et al. [38]	2021	observational (20 retro)	1	1	0	0.5	1	1	0	1	1	0	1	1	1	1	1	1	12.5	Low
Cerclage	Hulshoff et al.* [39]	2022	observational	1	1	0	0.5	1	0	0	1	1	0	1	1	1	1	1	1	11.5	Low
Cerclage	Marchand et al. [40]	2020	observational & case-controls	1	0	0	0.5	1	1	0	0.5	1	0	1	1	1	1	0	1	10	Critically low
Cerclage	Marchand et al. [41]	2022	RCT (1) & observational (42)	1	0	0	0.5	0	0	0	0.5	1	0	1	1	1	1	1	1	9	Critically low
Adnexal-ovarian	Cagino et al. [42]	2021	observational	1	0	0	0.5	1	1	0	1	0.5	0	1	1	1	1	1	1	11	Critically low
Adnexal-ovarian	Liu et al. [43]	2016	observational	1	0	1	0.5	1	1	0	1	1	0	1	1	1	1	0	1	11.5	Critically low
Adnexal-ovarian	Ye et al. [44]	2018	observational (9 retrospective)	1	0.5	0	0.5	1	1	0	1	1	0	1	1	1	1	1	1	12	Low
Cholecystectomy-biliary	Sedaghat et al. [45]	2017	observational	1	0.5	0	1	0	1	1	1	0	0	1	0	0**	1	1	1	9.5	Critically low
Total	-	-	-	18	4	2	10.5	14	12	2	16	11	0	14	9	10	12	10	16	7.2273***	-

Discussion

In the current review, we investigated primarily the safety and secondarily the outcomes of laparoscopic surgery during pregnancy, according to existing evidence from SRs and MAs. Furthermore, we scrutinized the methodological quality of pertinent evidence by means of the AMSTAR 2 instrument.

The most thoroughly investigated disciplines, according to the literature, have been LA, laparoscopic cervical cerclage, and laparoscopy for ovarian mass. Beyond the number of relevant reviews, these disciplines also bear the highest level of evidence given that they are the ones that are based on observational studies rather than case reports or case series. Notably, our search yielded only one MA that contained one RCT [41], whereas the rest of SRs and MAs included exclusively retrospective and prospective observational studies. Consequently, the first conclusion is that current practice is based on a lower-than-optimal level of evidence, which simultaneously signals the major limitation of our study. At the same time, we need to acknowledge the fact that designing and conducting RCTs that entail invasive interventions during pregnancy is a challenging task, as this would give rise to serious ethical, methodological, and practical concerns, given the widespread use of laparoscopy in current practice.

The second area of interest is the heterogeneous distribution of disciplines across the published literature. Appendectomy, cerclage, and adnexal pathologies are relatively overrepresented in existing reviews. This can only partially be attributed to the prevalence of relevant pathologies. For example, biliary colic and cholecystitis also constitute common entities during pregnancy, nevertheless most clinicians would opt for conservative management and postponement of definitive surgical intervention until after delivery, unless the safety of the mother and the fetus are severely compromised and placed at risk, despite the existence of evidence supporting the opposite [47,48]. The bottom line is that SRs and MAs on laparoscopic cholecystectomy during pregnancy are lacking, as is the case for emerging disciplines, such as long-term complications related to anatomic and physiologic alterations following metabolic bariatric surgery (MBS).

Given the abundance of reviews on LA during pregnancy, this procedure could also serve as an “archetypal” modality for evaluating the safety of laparoscopy during pregnancy. Augustin et al. had already undertaken an overview of systematic reviews specifically focusing on the safety of LA back in 2020 [49]. Their research yielded four SRs [31-33,35], all of which have also been included in our analysis in addition to more recent ones. Their conclusions validate our results in that the SRs suffered critically low methodological quality, while the heterogeneity in methodology and inclusion criteria prohibit the generalization of conclusions as they yield inconsistent results. 

In 2022, the European Association of Endoscopic Surgery (EAES) published a guideline that was based upon an updated in-house systematic review and meta-analysis [50]. The authors suggest that LA is performed before the 20th week of gestation or when the uterus is below the level of the umbilicus, whereas past this chronological and anatomical landmark, the choice between OA or LA should be based upon the expertise and preference of the surgeon. In any case, the authors recommend that pneumoperitoneum should be established in an open fashion. Besides, these suggestions bear a weak level of recommendation, given that pertinent literature suffers the same limitations as the ones described earlier. Furthermore, we could comment that these recommendations are rational and promote safety with respect to surgical technique. However, one should be cautious in any attempt to extrapolate these recommendations to other pathologies, since the natural course and medium- and long-term complications of the disease are not shared between appendicitis and other benign entities, including (but not limited to) cholecystitis, biliary calculi, complicated abdominal wall hernias, and adnexal torsion. Certainly, other entities with different biological behavior, like cancer, warrant an entirely different approach and management, though available evidence on laparoscopic management during pregnancy is scarce, largely because of the rarity of visceral malignancies coinciding with pregnancy [51-53]. 

The management of surgical emergencies during pregnancy following MBS is another topic of interest, which is predicted to increase over the following years, owing to the increase in metabolic bariatric operations worldwide [54,55], the improvement of female fertility following MBS [56,57], and the reduction of visceral fat that may serve as a predisposing mechanism for the formation of internal hernias [58,59]. Typically, the acute abdomen after MBS is treated laparoscopically; however, special considerations that apply to pregnancy should be addressed effectively before deciding the surgical access [60].

Our study has several limitations. As mentioned earlier, the included reviews mostly contained observational studies and case controls, which attenuates the strength of consequent evidence. Furthermore, the inclusion of a diverse spectrum of operations and pathologies increases the heterogeneity of the study. This effect might be of particular importance when combining gynecological and non-gynecological entities because the former might have direct implications on the outcome of pregnancy which might act as confounders for the effect of laparoscopy as an independent factor. In the same vein, we have included some studies that investigated the role of robotic-assisted surgery along with laparoscopy which might also contribute to the increase of heterogeneity. However, we found it appropriate to investigate all methods of minimally invasive surgery currently available. Last but not least, the overlap among primary studies within the included meta-analyses was very high, according to the GROOVE tool. This means that the included reviews largely depend on the same sources for drawing their conclusions.

## Conclusions

Based upon current low-quality, highly overlapping evidence, laparoscopy during pregnancy seems to be a safe approach. Nevertheless, surgical expertise, the natural history of each surgical disease individually, the age of pregnancy, and practical issues need to be taken into account and effectively addressed before proceeding with laparoscopy during pregnancy.

## Appendices

Appendix A: search strategy

Search words: laparoscop*, pregnancy; Filters: Meta-Analysis, Systematic Review; Sort by: Most Recent

("laparoscop*"[All Fields] AND ("pregnancy"[MeSH Terms] OR "pregnancy"[All Fields] OR "pregnancies"[All Fields] OR "pregnancy s"[All Fields])) AND (meta-analysis[Filter] OR systematicreview[Filter])

Translations

pregnancy: "pregnancy"[MeSH Terms] OR "pregnancy"[All Fields] OR "pregnancies"[All Fields] OR "pregnancy's"[All Fields]

Appendix B: excluded review studies

**Table 2:** List of excluded review studies with reasoning.

Year	Country	DOI (or PMC, in case of missing DOI)	Discipline	Full text retrieved	Reason(s) for exclusion
2010	Belgium	10.1016/j.bpobgyn.2009.08.001	Gynecologic cancer	Yes	Narrative review
2015	Belgium	10.1016/j.bpobgyn.2015.02.006	Cancer	Yes	Narrative review
2015	Hungary	10.1159/000437337	Renal cancer	Yes	Narrative review
2018	Greece, Cyprus	10.1016/j.jare.2018.02.006	Ovarian cancer	Yes	Narrative review
2019	Italy	10.1016/j.clgc.2019.05.025	Renal cell carcinoma	Yes	Narrative review
2014	Germany	10.1016/j.ejca.2013.12.020	Epithelial ovarian cancer	Yes	Narrative review
2015	Belgium	PMC4402440	Ovarian cysts and cancer	Yes	Narrative review
2014	Belgium	10.1007/s11912-014-0415-z	Gynecologic cancer	Yes	Narrative review
2019	USA	10.1016/j.ygyno.2020.03.015	Gynecologci cancer	Yes	Narrative review
2012	USA	10.1016/S0140-6736(11)60829-5	Gynecologic cancer	Yes	Narrative review
2015	India, UK	10.1016/j.bpobgyn.2015.10.015	Ovarian cysts and cancer	Yes	Narrative review
2012	Spain	10.1177/000313481207800316	Pheochromocytoma	Yes	Case report
2012	Italy	10.1007/s13304-013-0198-z	Uterine leiomyoma	Yes	Narrative review
2015	Italy	10.1097/GCO.0000000000000220	Uterine fibroids	Yes	Narrative review
2013	China	10.1159/000346334	Renal cell carcinoma	Yes	Narrative review
2021	China	10.1186/s12882-021-02318-w	Renal tumors	Yes	Narrative review
2019	UK	10.1016/j.ijsu.2019.09.016	Gallstone pancreatitis	Yes	Narrative review
2010	Turkey	Eastern J Med 2010;15:1-6	Nonobstetric surgery	Yes	Narrative review
2011	India	10.3909/riu0505	Nephrectomy for pyelonephritis	Yes	Case report & narrative review
2017	Portugal	Surg Technol Online 2017	Adnexal torsion	Yes	Narrative review
2020	USA	10.1016/j.fertnstert.2020.02.007	Laparoscopic cervical cerclage	Yes	Narrative review
2021	Italy	10.1177/0300891620909144	Adnexal masses	Yes	Narrative review
2014	France	10.1038/jp.2013.161	Acute pancreatitis	Yes	Narrative review
2013	UK	10.1016/j.surge.2013.11.022	Appendicitis	Yes	Narrative review
2014	USA	10.1097/GCO.0000000000000048	Adnexal masses	Yes	Narrative review
2011	USA	10.1016/j.ajog.2011.01.050	Adnexal masses	Yes	Narrative review
2017	Japan	10.1111/ases.12456	Appendectomy	Yes	Narrative review
2015	USA	10.1097/GRF.0000000000000088	Adnexal masses	Yes	Narrative review
2014	Brazil	10.1590/1806-9282.61.02.170	Appendicitis	Yes	Narrative review
2011	Greece	10.1016/j.ejogrb.2011.07.037	Acute pancreatitis	Yes	Narrative review
2015	Romania, Slovakia, Austria, Greece	10.1089/lap.2014.0624	Laparoscopic appendectomy	Yes	Narrative review
2020	Australia	10.1080/01443615.2020.1734781	Ovarian cysts	Yes	Narrative review
2011	Croatia	10.1097/MEG.0b013e328349b199	Acute pancreatitis	Yes	Narrative review
2015	USA	10.1055/s-0035-1549216	Adnexal masses	Yes	Narrative review
2015	Australia	10.1111/tog.12188	Appendicitis	Yes	Narrative review
2013	UK	10.4103/2006-8808.110249	Adnexal masses	Yes	Narrative review
2020	the Netherlands	10.1016/j.soard.2020.05.019	Small bowel intussusception	No	Full text could not be retrieved
2009	Thaiand	10.1002/14651858.CD005459.pub2	Benign ovarian tumor	Yes	Zero articles
2013	Thaiand	10.1002/14651858.CD005459.pub3	Benign ovarian tumor	Yes	Zero articles
2023	USA	10.1016/j.ajog.2022.11.1291	Adnexal masses	No	Full text could not be retrieved
2020	USA	10.7759/cureus.8959	Hernia	Yes	Narrative review
2018	Italy	10.1016/j.pan.2018.09.010	Pancreatic cystic tumors	Yes	Narrative review
2022	Korea	10.1080/01443615.2022.2107421	Fallopian tube torsion	No	Full text could not be retrieved; narrative review
2016	Italy, USA	10.4158/EP151009	Catecholamine-secreting tumors	No	Full text could not be retrieved
2020	USA	10.1097/GRF.0000000000000529	Appendicitis, cholecystitis	No	Full text could not be retrieved
